# Therapeutic implications for sphingolipid metabolism in metabolic dysfunction-associated steatohepatitis

**DOI:** 10.3389/fendo.2024.1400961

**Published:** 2024-06-19

**Authors:** Bruno Ramos-Molina, Joana Rossell, Alejandra Pérez-Montes de Oca, Eva Pardina, Idoia Genua, Marina I. Rojo-López, María Teresa Julián, Núria Alonso, Josep Julve, Didac Mauricio

**Affiliations:** ^1^ Group of Obesity, Diabetes & Metabolism, Instituto Murciano de Investigación Biosanitaria (IMIB), Murcia, Spain; ^2^ Group of Endocrinology, Diabetes & Nutrition, Institut de Recerca SANT PAU, Barcelona, Spain; ^3^ Centro de Investigación Biomédica en Red (CIBER) de Diabetes y Enfermedades Metabólicas Asociadas, Instituto de Salud Carlos III, Madrid, Spain; ^4^ Department of Endocrinology & Nutrition, Hospital Universitari Germans Trias i Pujol, Badalona, Spain; ^5^ Department de Biochemistry & Molecular Biology, Facultat de Biologia, Universitat de Barcelona (UB), Barcelona, Spain; ^6^ Department of Endocrinology & Nutrition, Hospital de la Santa Creu i Sant Pau, Barcelona, Spain; ^7^ Faculty of Medicine, University of Vic/Central University of Catalonia (UVIC/UCC), Vic, Spain

**Keywords:** steatotic liver, hepatic fibrosis, steatohepatitis, sphingolipids, inflammation, lipotoxicity, mitochondrial dysfunction

## Abstract

The prevalence of metabolic dysfunction-associated steatotic liver disease (MASLD), a leading cause of chronic liver disease, has increased worldwide along with the epidemics of obesity and related dysmetabolic conditions characterized by impaired glucose metabolism and insulin signaling, such as type 2 diabetes mellitus (T2D). MASLD can be defined as an excessive accumulation of lipid droplets in hepatocytes that occurs when the hepatic lipid metabolism is totally surpassed. This metabolic lipid inflexibility constitutes a central node in the pathogenesis of MASLD and is frequently linked to the overproduction of lipotoxic species, increased cellular stress, and mitochondrial dysfunction. A compelling body of evidence suggests that the accumulation of lipid species derived from sphingolipid metabolism, such as ceramides, contributes significantly to the structural and functional tissue damage observed in more severe grades of MASLD by triggering inflammatory and fibrogenic mechanisms. In this context, MASLD can further progress to metabolic dysfunction-associated steatohepatitis (MASH), which represents the advanced form of MASLD, and hepatic fibrosis. In this review, we discuss the role of sphingolipid species as drivers of MASH and the mechanisms involved in the disease. In addition, given the absence of approved therapies and the limited options for treating MASH, we discuss the feasibility of therapeutic strategies to protect against MASH and other severe manifestations by modulating sphingolipid metabolism.

## 1 Introduction

Metabolic-associated steatotic liver disease (MASLD, formerly known as non-alcoholic fatty liver disease, NAFLD) (1) is defined as the pathological accumulation of lipids in the form of lipid droplets in more than 5% of hepatocytes (steatotic liver) and the presence of at least one cardiometabolic risk factor (2). MASLD has emerged as the most prevalent chronic liver disease, affecting approximately 30% of the global population (3). Surpassing viral hepatitis in incidence, MASLD has become a significant public health concern with a steadily growing impact on healthcare systems worldwide. MASLD represents a continuum of chronic liver disorders, ranging from simple steatosis to more advanced stages, such as metabolic dysfunction-associated steatohepatitis (MASH, formerly known as NASH) (1). MASH, characterized by steatosis, lobular or portal inflammation, and hepatocellular ballooning, may progress to liver fibrosis, cirrhosis, and ultimately hepatocellular carcinoma (HCC).

According to a recent meta-analysis involving 26,738 patients with MASLD, it has been estimated that around 30% of patients developed MASH after less than five years (4). Notably, fibrosis progression can occur in both MASLD and MASH, but the incidence rate of hepatic fibrosis is higher in MASH patients (5). In addition to fibrosis, MASLD patients have been reported to develop type 2 diabetes mellitus (T2D) or impaired glucose tolerance in the long term (6). Furthermore, long-term follow-up evaluation of MASLD patients showed that MASH patients have increased liver-related mortality compared with those without MASH, especially when T2D is present (7).

MASH is often diagnosed incidentally as it is considered a clinically silent disease (8). Currently, predicting the course of the disease in a specific manner is challenging due to the lack of low-cost and easily accessible diagnostic tools for routine monitoring of individuals at high risk of progression. While there have been advancements in MASLD concerning the diagnosis of steatosis and fibrosis (9, 10), liver biopsy continues to be the ‘gold standard’ for MASH diagnosis due to the absence of thoroughly validated biomarkers for diagnosis, prognosis, and monitoring of disease progression. However, its use is limited by invasiveness, rare but potentially fatal complications, sample variability, and high cost. Additionally, given the high prevalence of MASH in the population, widespread evaluation with liver biopsy is neither practical nor recommended (11). Therefore, there is an urgent need to identify new non-invasive and reliable biomarkers that can serve as alternatives to liver biopsy for the diagnosis and prognosis of MASH.

Whilst the metabolic drivers contributing to the transition from simple steatosis to MASH remain partly elusive (12, 13), recent investigations suggest that lipotoxicity may play a vital role in the progression of MASLD, as it can boost inflammation and promote hepatic fibrogenesis (14, 15). Derangements of lipid metabolism stands out as a major factor contributing to MASLD and advanced active form of this condition (16). In this regard, accumulating experimental evidence supports that lipotoxicity can result from the accumulation of different potentially cytotoxic lipid intermediates derived from sphingolipid metabolism (17).

The use of omics approaches represents a potent strategy to uncover hidden, distinct signatures of biomarkers derived from lipid metabolism related to liver disease. Sphingolipids, which can be divided into three different structural categories, i.e., sphingoid bases and derivatives, ceramides, and complex sphingolipids (18), have unique roles as signaling molecules (16). As such, differentially-expressed sphingolipids can contribute to the cellular reprogramming relevant to the development of MASLD and contribute to its progression to MASH (16). Noteworthy, some ceramide and other complex sphingolipid species are increased in obesity and T2D, posing them as potential candidates to direct metabolic drivers of hepatocellular injury (17). In this context, a growing body of recent evidence also supports the notion that some commonly used anti-diabetic therapies (e.g., thiazolidinediones, metformin, glucagon-like peptide-1 receptor agonist (GLP-1 RA)) may exert protection against hepatic lipotoxicity. This protective effect is achieved by reducing the synthesis of reactive sphingolipid species in target non-adipose tissues, including the liver, thereby unraveling potentially novel benefits of current therapies.

In this review, we will mainly discuss [i] the alterations in sphingolipid metabolism possibly involved in MASLD pathogenesis, with particular emphasis on those related to its progression to MASH, and [ii] the impact of current and potentially novel therapies that target sphingolipid metabolism for MASH treatment.

## 2 Intracellular metabolism and cellular actions of sphingolipids

**Figure 1:**
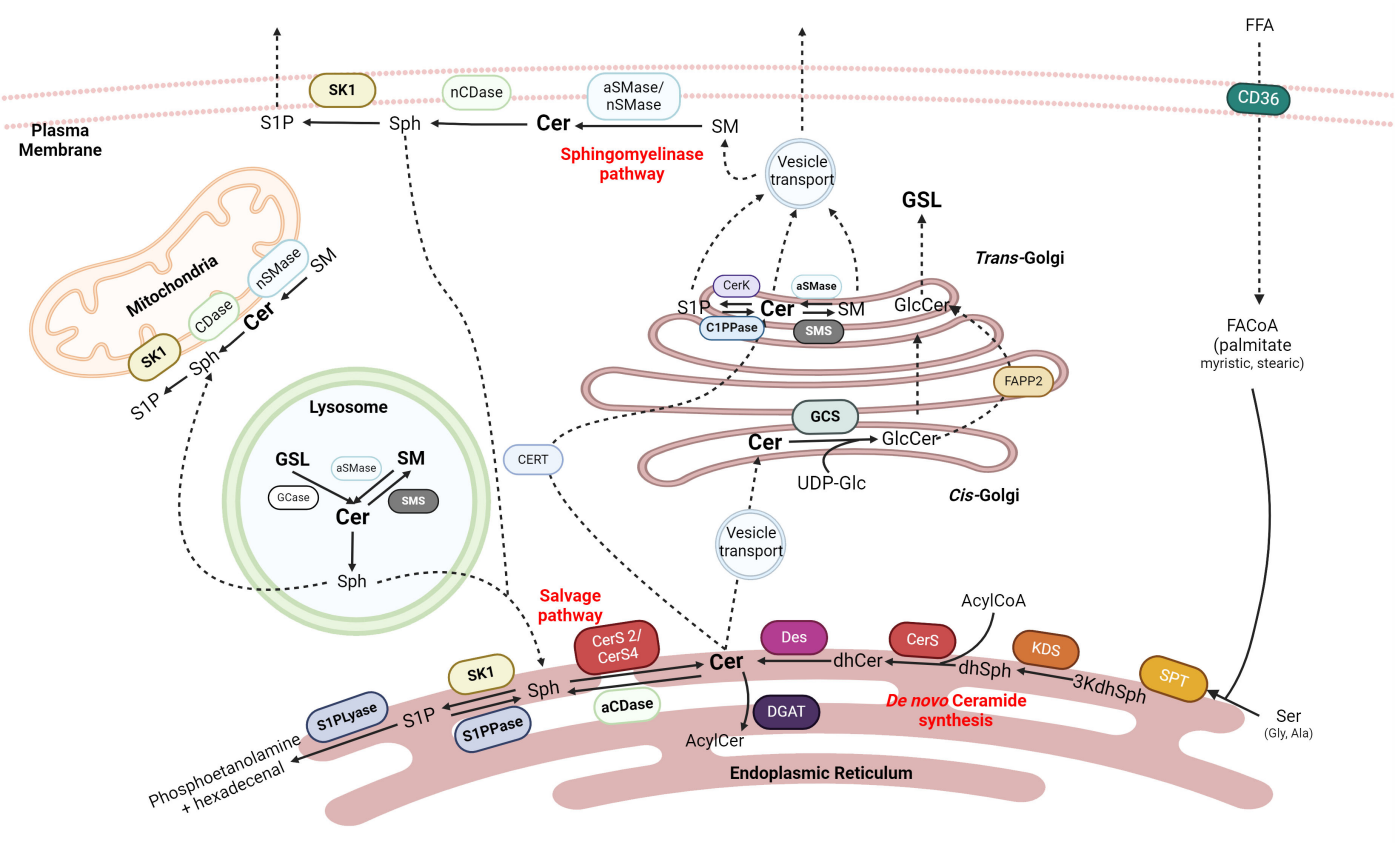
Sphingolipids synthesis. Ceramide (Cer) is one of the central precursors of Sphingolipids. Cer can be obtained in the ER by *de novo* synthesis with the condensation of palmitoyl-CoA and serine or by salvage pathways from more complex sphingolipids in the mitochondria, lysosome, ER, or plasma membrane. Cer can be exported to Golgi to form Glucocerebrosides (GLC) or Sphingomyelin (SM). Cer can produce Sphingosine (Sph) in the plasma membrane that can be recycled again into Cer and sphingosine-1-P (S1P), which is exported out of the cell. SPT, serine palmitoyltransferase; 3kdhSph, 3-ketodihydrosphingosine; KDS, 3-ketoreductase; dhSph, dihydrosphingosine; CerS, ceramide synthase; Des, desaturase; aCDase, acid ceramidase; nCDase, neutral ceramidase; S1PPase, sphingosine 1 phosphate phosphatase; S1PLyase, sphingosine 1 phosphate lyase; GCase, glucosylceramidase; GCS, glucosylceramide synthase; aSMase, acid sphingomyelinase; nSMase, neutral sphingomyelinase; SMS, sphingomyelin synthase; FAPP2, four-phosphate adaptor protein 2. Created with BioRrender.com.

Sphingolipids, one of eukaryotes’ most complex bioactive lipids, are ubiquitous. Apart from their structural and/or energetic functions, they regulate several cellular processes (19, 20). Sphingolipids are derivates of long-chain aliphatic amino alcohols, with a backbone of sphingoid bases. In mammals, these sphingoid bases are mostly derived from dihydrosphingosine (also known as sphinganine) or sphingosine, and they actually include hundreds of compounds (21). Sphinganine and sphingosine differ by their synthesis pathway (**Figure 1**). While sphingosine is produced through the salvage pathway, using ceramide as a substrate, sphinganine is synthesized in the *de novo* biosynthesis pathway as a result of the condensation of serine and palmitoyl-CoA. Sphingoid bases can be N-acylated to form ceramides; phosphorylated to form sphingomielin or glycosilated to give rise to more complex lipids, the glycosphingolipids, i.e. cerebrosides, globosides or gangliosides (22).

Historically, sphingosine was the first sphingolipid described by Thudichum in 1884 (23). It was structurally characterized by Herb Carter in 1947, who defined “lipids derived from sphingosine as sphingolipids” (24). Currently, ceramides are considered critical intermediates in the metabolism of sphingolipids (22, 25).

### 2.1 Intracellular metabolism of sphingolipids

There are elegant reviews where the biosynthesis of sphingolipids has been described in depth (22, 25, 26). In summary, ceramides can be synthesized in *de novo* and salvage pathways (**Figure 1**).

The *de novo* biosynthetic pathway begins in the cytosol of the endoplasmic reticulum (ER) with the condensation of a saturated acyl-CoA with an amino acid by serine palmitoyltransferase (SPT), the key regulatory enzyme of the pathway, giving 3-ketodihydrosphingosine (3-KDHS). While palmitate and serine are the canonical substrates for SPT, depending on the enzyme’s isoform, various fatty acids (stearic or myristic acid) and amino acids (alanine or glycine) can also be used, resulting in the formation of atypical sphingoid bases that increases the diversity of sphingolipids (27).

After the reduction of 3-KDHS, dihydrosphingosine is obtained. The ceramide synthase (CerS) family, through N-acylation and subsequent addition of a double bound by desaturase 1 or 2 (Des 1 and Des2, respectively), generates a plethora of more than 200 different types of ceramides (28). While there are six mammalian CerS, only Cer2 - with a preference for fatty acyl CoA C22 to C24 - and CerS4 - with a preference for fatty acyl CoA C18 to C20 - have been expressed in the liver (29). Their substrate specificity determines the fatty acid composition of sphingolipids (30).

The salvage pathways are those that obtain ceramide from more complex sphingolipids. One such pathways is the sphingomyelinase pathway (25), where sphingomyelin (SM) is hydrolyzed by the action of sphingomyelinases (SMases). Another option is the deacylation of ceramide to sphingosine by ceramidases (CDases); this sphingosine can be reacylated by CerS again. In humans, five different CDases have been described which have been named according to their optimum catalytic pH: alkaline CDase (ACER 1, 2 and 3), acid CDase (ASAH1), and neutral CDase (ASAH2) (31). The last option involves the degradation of glycosphingolipids in late endosomes or lysosomes by hydrolases that remove the added glycans until ceramide is obtained again (25).

Ceramides can be further modified to obtain many other complex bioactive lipids. Still in the ER, galactose can be transferred into the C1 ceramide’s backbone by ceramide galactosyl transferase (CGT), obtaining galactosphingolipids that could become galactocerebrosides (32). For the formation of other glycosphingolipids, such as globosides or gangliosides, ceramides must be transported into the trans-Golgi either by the phosphatidylinositol-four-phosphate adaptor protein 2 (FAPP2) or by vesicular transport, respectively (33); and finally suffer glycation with UDP-glucose by the glucosylceramide synthase (GCS) (34, 35).

On the other hand, CERT-transported ceramides are converted to SM by sphingomyelin synthase 1 (SMS1) in the cis-Golgi by the transference of a phosphorylcholine group from phosphatidylcholine (33). Finally, ceramides can also be phosphorylated by ceramide kinase (CerK), giving ceramide 1P in the trans-Golgi (36, 37), or acylated at the 1-OH position by diacylglycerol O-acyltransferases (DGATs) in ER (38, 39).

Noteworthy, sphingosine generated by CDases can be phosphorylated by sphingosine kinases 1 (cytosolic) and 2 (mitochondrial and nuclear) (SK1 and SK2, respectively), obtaining sphingosine-1-phosphate (S1P) (40). S1P can serve as an intracellular signal transduction molecule via the G-coupled receptors S1PR1–5, which are expressed in many tissues and modulate signals by RAS-GTP, phosphoinositide-3-kinase (PI3K), phospholipase C (PLC) and Rho (41). Otherwise, S1P can enter the irreversible catabolic pathway of ceramides using S1P lyase, which cleaves the molecule into an ethanolamine phosphate and a fatty aldehyde (hexadecenal), which in turn could be further metabolized to enter the energetic lipid metabolism (42).

### 2.2 Cellular actions of sphingolipids

As stated above, sphingolipids are involved in many cellular processes. Among the latter, the most important are apoptosis and autophagy. Ceramides have been described mainly as proapoptotic drivers. On the one hand, they have been described as activators of the JNK signaling pathway (43). Besides, ceramides, and Bcl2-family pro-apoptotic proteins (Bax and Bak), can form barrel-like channels in the outer layer of mitochondrial membranes (29), guiding cell to death by permeabilization of the organelle. Surprisingly, there is also evidence that long-chain polyunsaturated ceramides can also be anti-apoptotic (44, 45). Phosphorylated sphingolipids such as S1P, C1P, or ganglioside GD3 have been related to the autophagosome formation for survival metabolism (29). S1P is also a potent inducer of proliferation and cell survival (46). Of note, the anti-inflammatory adipokine Adiponectin has been related to the upregulation of CDase, increasing S1P concentration (47).

Besides their intervention in programmed cell death, ceramides are also related to mitochondrial dysfunction. For example, the C16:0 ceramides (Cer16) derived from CerS6 activity interfere in the mitochondrial fission (45). Moreover, ceramides also induce oxidative stress in hepatic mitochondria as their accumulation promotes the production of the ganglioside GD3 in the ER, which induces the production of superoxide anion (48).

Considering the aforementioned actions, it is unsurprising that sphingolipids also play a role in modulating inflammation. Ceramides can induce inflammation by the activation of the NF-κB – TLR4 pathway (49) and the NLRP3 (NOD-, LRR, and pyrin domain-containing protein 3) inflammasome (50) resulting in an increase of proinflammatory cytokines such as IL-1β or IL-18 (51). S1P generated by SK1 has also been described to act through the NF-κB pathway by S1PR1–3 (52). In turn, the secretion of proinflammatory cytokines stimulate several key enzymes of the sphingolipid metabolic pathway (SPT, CerS1, CerS2 and CerS6, SMases) (49, 53).

## 3 Alterations of sphingolipid metabolism in MASH pathogenesis

Hepatic inflammation, a diagnostic characteristic of MASH, is triggered by several factors (54). Among these factors, the development and progression of MASLD to MASH has been linked to disturbances in hepatic sphingolipid metabolism, as revealed using metabolomics approaches (55–57). The relationship between increased hepatic inflammation and ceramide content is intricate and bidirectional (48). As such, the *de novo* synthesis of ceramides may be upregulated via toll-like receptor (TLR)-dependent mechanisms, whereas ceramides can exacerbate hepatic inflammation by activating TLR and inflammasome signaling pathways.

The hepatic content of ceramides is frequently increased in experimental MASLD and may mediate its progression to MASH (58–60). Experimental studies support the notion that increased hepatic ceramides and upregulation of target genes encoding key enzymes involved in their synthesis, including CerS, are related to diet-induced MASH/hepatic fibrosis in mice (61, 62). Therefore, targeting CerS isoforms, which are responsible for the synthesis of distinct ceramide species (48), may become a potential therapeutic approach to reduce ceramide synthesis. Consistently, genetic causes of CerS2 deficiency, the dominant form in the liver that preferentially synthesizes very-long-chain (Cer22/Cer24/Cer24:1) ceramide species, results in concurrent reductions in these ceramides species in the liver (63). Additionally, the pharmacological blockage of *de novo* synthesis of ceramides using myriocin, a serine palmitoyl transferase inhibitor, protects against hepatic ceramide accumulation and can prevent the development of MASH in rats (64). Intriguingly, the synthesis of long-chain Cer16 was upregulated in the above-mentioned genetic setting of CerS2 deficiency, and therefore the susceptibility to diet-induced steatohepatitis (63). The underlying mechanism responsible for such increase in C16-ceramides was not further investigated, but, at least in part, it could be likely compensatory, thereby shedding doubts in considering CerS as a molecular target in future therapies.

Although the evidence in humans is somewhat limited, direct determination of sphingolipids has been addressed in hepatic biopsies only in a few studies for obvious ethical reasons. Accumulating clinical studies show that circulating and hepatic ceramides are strongly associated with MASH (65–69). Of note, hepatic total ceramides were particularly higher in obese subjects with steatohepatitis compared with those with steatosis or without MASLD (69). Alongside ceramides, the sphingolipid profile has also been characterized by concurrent elevations in the hepatic content of their direct precursors, the dihydroceramides (70). Consistently, the hepatic content, of dihydroceramides (16:0, 22:0, and 24:1) and lactosylceramides are increased in subjects with steatohepatitis (69). In agreement with this, the hepatic levels of dihydroceramides were increased along with the histological severity in patients with MASLD (71, 72). More specifically, hepatic levels of Cer(d18:0/20:0) and Cer(d18:0/24:1) have been shown to be significantly elevated in subjects with MASH compared with those with simple steatosis (72). In addition, several long-chain ceramides, dihydroceramides, or 1-deoxy-dihydroceramides have also been reported to display an overlap between differentially-expressed lipids in both the liver and peripheral circulation, thereby unveiling their potential as biomarkers for diagnostic purposes (67, 71). These findings indicate that a high level of circulating ceramides is a common feature of MASH in both adult and pediatric patients.

A distorted sphingolipid metabolism is directly linked to hepatic insulin resistance, a well-known hallmark of MASLD (20, 69). Insulin-deficient signaling states directly correlate with concurrent elevations of some sphingolipid species in subjects with MASH, suggesting that these lipids may play a role during progression from simple steatosis to MASH. Supporting this notion, in a recent study made on people with severe obesity undergoing bariatric surgery, the hepatic content of dihydroceramide, which is considered a marker of *de novo* synthesis of ceramides, and ceramides were markedly increased in subjects with higher HOMA-IR (68). Similar results were also reported in an independent study, where the total ceramide concentration was positively correlated with HOMA-IR and insulin levels in obese children with MASLD (73). Additionally, the hepatic content of dihydroceramides and ceramides was markedly increased, along with that of free fatty acids and triglycerides, in subjects with significantly higher insulin resistance (68). In another independent study, circulating total ceramides were inversely correlated with whole-body insulin sensitivity in subjects with obesity and insulin resistance (with and without MASLD/MASH) who were eligible for bariatric surgery (69). These findings reinforce the contribution of impaired insulin signaling to dysregulated sphingolipid metabolism and its relationship with MASH.

There are several possible mechanisms by which sphingolipids can modulate insulin signaling: a) ceramides are described to impair insulin activation of Akt/PKB altering its translocation (via PKCζ or protein phosphatase 2 a -PP2A); b) alternatively-phosphorylated RNA-dependent protein kinase (PKR) by ceramides would prevent the binding of IRS1 to PI3K; and c) using changes in plasma fluidity (20, 48). Glucosylceramides and gangliosides (especially GM3) are also involved in insulin resistance, as GM3 interferes directly with IRS1 (74). The results of S1P insulin modulation are controversial. When S1P is bound to apolipoprotein (apo)M, it enhances insulin signaling via Akt-pathway activation through S1PR1 and S1PR3, whereas S1P bound to albumin would interfere with insulin sensitivity through SP1R2 (75).

The hepatic ratio of long-chain ceramides vs. very long-chain ceramides has been described as a marker of metabolic diseases, including diabetes (63, 68). Furthermore, the excess of hepatic non-esterified fatty acid influx, the inflammatory processes (that is, elevated TNF-alpha and IL1), and the oxidative stress altogether promote the SPT activation, increasing the *de novo* ceramide synthesis and the SMase activation, increasing the SMase pathway (26, 76). On the other hand, the hepatic S1P produced from the excess of palmitate activates local stellate cells by signaling through S1PR3. It increases myofibroblast migration, establishing a possible relationship with fibrosis (77).

Lipidomic analysis may also enable the identification of changes related to the degree of hepatic fibrosis in the context of MASH. Specifically, the lipid composition of livers from obese patients with MASH significantly differed depending on the extent of hepatic fibrosis (78). Sphingomyelin levels, particularly those of SM (35:0) and (37:0) species, were significantly reduced in the livers from MASH patients with significant fibrosis. In contrast to ceramides, sphingomyelin content favorably influences cell survival (79). Indeed, the balance between sphingomyelin and ceramides is considered a predictor of cell survival and proliferation (79). Accordingly, an increased ceramide-to-sphingomyelin ratio due to reduced sphingomyelin in subjects with MASH and significant hepatic fibrosis could suggest that the sphingolipid profile may be worsened compared to those with mild hepatic fibrosis (78).

## 4 Experimental/clinical evidence of therapies targeting sphingolipid metabolism and its impact on MASLD/MASH

**Figure 2:**
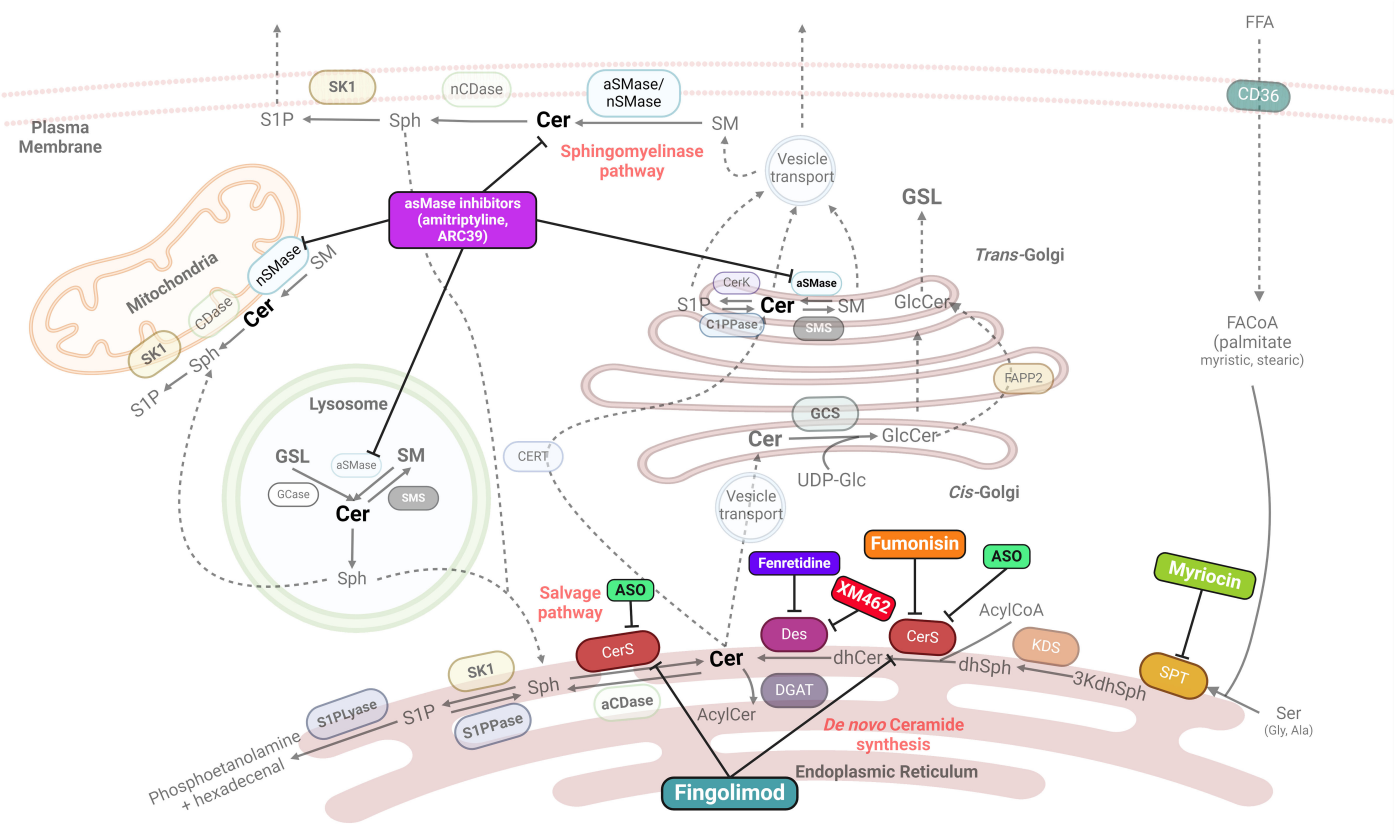
Main known drug action targets on sphingolipid synthesis. Many potential therapies for MASH/MAFLD target key enzymes involved in sphingolipid synthesis, particularly ceramides. SPT, serine palmitoyltransferase; 3kdhSph, 3-ketodihydrosphingosine; KDS, 3-ketoreductase; dhSph, dihydrosphingosine; CerS, ceramide synthase; Des, desaturase; aCDase, acid ceramidase; nCDase, neutral ceramidase; S1P, sphingosine 1 phosphate; S1PPase, sphingosine 1 phosphate phosphatase; S1PLyase, sphingosine 1 phosphate lyase; GCase, glucosylceramidase; GCS, glucosylceramide synthase; aSMase, acid sphingomyelinase; nSMase, neutral sphingomyelinase; SMS, sphingomyelin synthase; FAPP2, four-phosphate adaptor protein 2; ASO, Antisense oligonucleotides. Created with BioRrender.com.

Due to the role of sphingolipids, and particularly ceramides, in inflammation and MASH development, sphingolipid metabolism is one of the potential targets to focus on for MASH treatment strategies. Several candidate compounds are currently known to favorably influence sphingolipid metabolism, thus becoming therapeutic drugs (**Table 1**). The sites of action for each specific drug are illustrated in **Figure 2**. Nevertheless, these compounds are at different stages of research. Several antidiabetic drugs have a beneficial effect on MASH through their action on sphingolipid metabolism. This section summarizes the current potential benefits of MASH therapies targeting sphingolipid metabolism.

**Table 1:** Drugs targeting sphingolipid metabolism with a potential therapeutic role on MASH*.

Compound	Therapeutic target	Experimental/ clinical model	Drug administration effects
Myriocin	SPT inhibitor	Rats	Myriocin was administered for 7 days by intraperitoneal injections (0.3 mg/kg of body weight). Myriocin reduced SM content in liver, but increased S1P (80).
		Rats	Myriocin was administered intraperitoneally every other day for 12 weeks (0.3 mg/kg of body weight). Reduced plasma glucose, reduced ceramide, sphinganine, and SM liver content. Increase in S1P. Reduction in liver DAG and TAG content. Reduced inflammatory cell aggregates (64).
		Rats	Myriocin was administered every other day by gavage for 8 weeks (0.3 mg/kg of body weight). Improved lipid profile and decreased serum transaminases. Corrected the expression pattern of fatty acid metabolism associated genes (65).
		Mice	Myriocin was administered by daily oral gavage for 12 weeks (0.3 mg/kg of body weight). Down-regulated the expression of MCP-1 and its receptor CCR2. Decreased pro-inflammatory Ly-6c high monocytes (81).
		Rats	Myriocin was administered intraperitoneally on alternate days, 3 times per week, for 5 weeks (0.3 mg/kg of body weight). Improve the affectations cause by alcohol on fatty acid lipid profile on liver (82).
		Mice	Cordyceps powdered sample was administered orally mixed with diet (19.7 mg/kg, containing 4–6 nmol/g myriocin). Reduced ceramide levels. Increase in energy expenditure (increase in beiging/browing of adipose tissue). Improved glucose homeostasis and resolved hepatic steatosis (83).
		*In vitro*	Cells were treated with 10 μM myriocin 16 h before collecting supernatants. Reduced the pro-inflammatory activity of intrahepatic Cholangiocarcinoma-derived extravesicular cells (84).
Fingolimod	S1P antagonist	Mice	Fingolimod was administered intraperitoneally for 2 weeks (1 mg/kg of body weight). Reduction of steatosis, liver injury, and inflammation (85).
		Mice	Fingolimod was administered via oral gavage three times a week for 16 weeks (0.3 mg/kg of body weight). Reduction in hepatic sphingolipid levels (ceramides, monohexosylceramides, and sphingomyelins). Reduced steatosis but no significant changes in hepatic inflammation, hepatocyte ballooning, NAFLD activity score, or fibrosis. Significant reductions in the expression of [i] CXCL10, also known as interferon γ-induced protein 10, a known chemoattractant for monocytes/macrophages, T-cells, and dendritic cells, and [ii] CCL5, also known as RANTES (regulated on activation, normal T-cell expressed and secreted), that plays an active role in the recruitment of leukocytes (86).
		*In vitro*	HepG2 cells were treated with various concentrations of fingolimod (0, 10, 100, 200, 500, and 1000 μM) for 24 h. Binding to S1PR3 which activates sequentially Gq, PI3K, and mTOR leading to an increase in SREBP expression and PPARγ activation (87).
Fumonisin B1	CerS Inhibitor	Rats	Administration FB1 at different concentrations over approximately 2 years (0.8 – 1.6 mg/kg of body weight). Hepatotoxic effects of FB1 (88).
		Mice	FB1 was administered orally mixed in the drinking water daily for 3 weeks (10 mg/kg body weight). Decreased body weight, blood fasting glucose, hepatic steatosis, and liver weight, but increased plasmatic transaminases. FB1 in obesity led to severe hepatic inflammation (89).
		Mice	FB1 was administered intraperitoneally every other day for 2 weeks 2.25 mg/kg of body weight). Resveratrol has protective effects against FB1 liver toxicity (90).
ASO	CerS Inhibitor	Mice	Antisense oligonucleotides targeting CerS6 were administered intraperitoneally 3 times (day 1, 4 and 8) (5 mL/kg body weight). Decreased body weight, plasmatic and liver ceramide content. Improved glucose homeostasis and decreased liver triglyceride content (91).
ASM inhibitor	Imipramine	Mice	Imipramine was administered intraperitoneally daily for 2 weeks (10 μg/g of body weight). Prevention of steatosis, reduced hepatic ceramides, and reduced activation of stress kinases. Improved blood glucose tolerance (92).
	Knock-out	Mice	Not progression to MASH (93).
	ARC39	*In vitro*	L929, HepG2, and B16F10 cells were treated with ARC39 at 1 mM. Reduction in total ceramides, toxicity after 48h (94).
	Amitriptyline	Mice	Amitriptyline was administered intraperitoneally every day for 10 weeks (0.5 mg/mouse). Inhibition of hepatic steatosis. Suppressed SP1 and ceramide formation, reduction of proinflammatory cytokines, suppressing hepatic inflammation (95).
		Mice	Amitriptyline was administered intraperitoneally every day for 6 weeks (2.5 mg/kg of body weight the first 4 weeks, and 5.0 mg/kg of body weight the last 2 weeks). Reduced hepatic levels of ceramides, protection from steatosis and MASH (96).
FXR agonists	Gly-MCA	Mice	Gly-MCA was orally administered every day for 8 weeks (10 mg/ kg of body weight). Reduced lipid accumulation, inflammatory response (in injury markers, ALT and AST), and collagen deposition in NAFLD and NASH models. Lower liver endoplasmic reticulum stress and proinflammatory cytokine production (97).
	Vonafexor	Human	Vonafexor was orally administered every day (100, 200, or 400mg). Reduced liver steatosis and showed an improvement in imaging biomarkers of fibrotic steatohepatitis after only 12 weeks of treatment (98).
DES1 deletion	Fenretinide	*In vitro*	RAW 264.7 cells were treated with Fenretinide (5 μM). Fenretinide decreased ceramide level and inhibited the release of proinflammatory cytokines (99).
		Mice	Fenretinide was orally administered at 0.04% mixed with a High-fat diet. Decrease in ceramides decrease in gene expression of IL-6, IL-1B, IL-10 (100).
	XM462	*In vitro*	C38 human bronchial epithelial cells were treated for 14h with XM462 (20 μM). Protection against inflammation and oxidative stress in lung cells exposed to smoke (101).
	Gene model	Mice	Resolved hepatic steatosis and insulin resistance (102).
Other approaches	Dietary fats	Human	Reduced circulating FA containing sphingolipids. Effect on T2D less clear (103).
	Predimed trial	Human	Reduction of circulating ceramides (103).
	Scoparone	Mice	Decrease in ceramides and other sphingolipids altered due to HFD in a MASH model (104).

ALT, alanine aminotransferase; AST, Aspartate transaminase; CCR2, monocyte chemoattractant protein-1 receptor; CerS, ceramide synthase; DAG, diacylglycerol; FA, fatty acids; FB1, Fumonisin B1; HFD, high-fat diet; Ly6c1, lymphocyte antigen 6 family member C1; MASH, metabolic dysfunction-associated steatohepatitis; MCP-1, monocyte chemoattractant protein-1; MASLD, metabolic associated steatotic liver disease; PI3K, Phosphoinositide 3-kinases; SM, Sphingomyelin; S1P, sphingosine-1-P; T2D, diabetes mellitus type 2; TAG, triglycerides.

*When indicated, the current terms MASH/MASLD that appear in this table were used instead of the traditional terms NASH/NAFLD.

### 4.1 Drugs known to act on different molecular targets involved in sphingolipid metabolism

#### 4.1.1 Myriocin

Myriocin is a compound originally isolated from a fungus commonly used in traditional Chinese medicine. This molecule inhibits the activity of serine-palmitoyl transferase (SPT), the rate-limiting enzyme in SM synthesis (105). This drug has been chiefly used in different *in vivo* models involving liver injury, with positive effects against MASLD and MASH. In this context, myriocin has been shown to reduce liver steatosis inflammation and overall liver injury in obese mice fed a high-fat diet (65, 85). Consistently, the treatment of streptozotocin-induced diabetic rats (80, 85), with myriocin successfully ameliorated the hyperglycemia and protected against hepatic steatosis in treated rats. Remarkably, this favorable phenotype was accompanied by a concomitant reduction in the hepatic content of ceramides, SM, and sphinganine but resulted in an increased hepatic content of S1P (64). Moreover, inflammatory cell aggregates were also reduced, showing that myriocin positively affected hepatic inflammation (64). Additionally, myriocin has been also shown to be hepato-protective in a mouse model of alcoholic liver disease. This was revealed by significantly lessening the hepatic fatty acid lipid profile after administering this compound (82). However, despite such positive results, further research is probably needed regarding using of myriocin in humans. In a recent article, Li et al. (83) used commercially available extracts derived from the fungus Cordyceps, also used in traditional Chinese medicine, as a myriocin source to treat mice fed a high-fat diet. These extracts reduced circulating ceramide levels, improved glucose homeostasis, and resolved hepatic steatosis in treated mice. Interestingly, myriocin-treated mice also showed an increase in the beiging/browning of adipose tissue, leading to increased energy expenditure, which partially prevented the obesogenic effects of the high-fat diet (83). Moreover, genetic models have provided important information on the mechanism behind the anti-inflammatory effects of myriocin. In ApoE-/- mice, myriocin decreased pro-inflammatory monocytes down-regulated the expression of monocyte chemoattractant protein-1 (MCP-1) and CD36 in sections of atherosclerotic lesions and the circulating levels of MCP-1 receptor (CCR2) (81).

#### 4.1.2 Fingolimod

Fingolimod is a chemical derivative of myriocin. In contrast to myriocin, fingolimod has lower toxicity and has been approved for human use for treating multiple sclerosis (106). This drug acts as an S1P antagonist, downregulating CerS2 (106, 107), thereby suggesting a mechanism of reduced very-long-chain ceramides. Mechanistically, fingolimod also binds to S1PR3, increasing SREBP expression and activating PPARγ through the activation of Gq, PI3K, and mTOR (87). In mice fed obesogenic diets, the administration of fingolimod has been associated with reduced steatosis, liver injury, and inflammation (85). However, the favorable impact of this drug on steatohepatitis was not proven in another diet-induced mouse model of MASLD (86). The reason for such controversy is not known but may at least in part be related to differences in experimental dietary inducers and the genetic background of the animal models used in each study (85, 86). Remarkably, despite having minimal effects on the expression of hepatic markers of inflammation in one of these two studies (86), its administration significantly decreased the gene expression of chemoattractants for different immune cells in the liver of treated mice (interferon γ-induced protein 10, i.e., CXCL10, and RANTES, i.e., CCL5), which are commonly dysregulated in liver fibrosis (108).

In a clinical setting, despite being approved for human use, fingolimod administration has also been linked to hepatotoxicity (109, 110), which complicates its feasibility to become a new treatment for MASLD/MASH.

#### 4.1.3 Ceramide synthase inhibitors

Another compound derived from fungi is Fumonisin B1 (FB1). This molecule reduces sphinganine formation through competitive-like CerS inhibition mechanisms (111, 112). FB1 can reduce hepatic steatosis and decrease body weight and fasting blood glucose in C57BL/6J mice fed a high-fat diet (89). Despite the positive effects, FB1 was associated with more inflammation, as shown by increased expression of inflammatory genes, and more inflammatory foci (89). Hepatotoxicity is a main drawback regarding the potential of FB1 to become a treatment candidate (88, 113). This toxicity also becomes much more pronounced with increased adiposity (89). Nevertheless, a recent study showed that these adverse effects can be mitigated when resveratrol is co-administered, as this polyphenol acts as an hepato-protector (90).

Antisense oligonucleotides (ASO) can be used to selectively inhibit the action of CerS (specifically CerS6). It results in decreased body weight, especially fat mass, and reduced plasmatic and liver ceramide content. Glucose homeostasis was generally improved, with reduction in insulin, glucose, and HOMA-IR indexes. Moreover, liver triglyceride content was also decreased (91).

#### 4.1.4 Specific inhibitors of acid sphingomyelinase

Another potential target in sphingolipid metabolism is the enzyme acid SMase (aSMase), which catalyzes SM hydrolysis to ceramides (114). aSMases are partly responsible for the upregulation of the inflammatory response seen in MASLD (95), thereby suggesting that therapies targeting this enzyme could protect against MASLD and/or MASH development. Supporting this notion, experimental data using genetically modified mice deficient in aSMase (aSMase (-/-)) developed steatosis when fed a Western diet. However, hepatic steatosis did not progress into MASH compared with the wildtype mice on the same diet (93). Consistently, the aSMase(-/-) mice fed a high-fat diet were resistant to the diet-induced hepatic ER stress, resulting in less hepatic inflammation (96). Although the hepatic content of ceramides was not directly measured in these previous studies, it could be suggested that these species would be reduced in the livers of aSMase-deficient mice.

Commercially available drugs, such as tricyclic antidepressants, have been shown to have inhibitory properties on aSMase activity and, thus, contribute to ameliorating steatosis or inflammation in hepatocytes (115). Amitriptyline is one of the possible candidates. In a study using an LDL receptor-deficient mice model to evaluate the role of aSMase in the inflammatory process, MASH and atherosclerosis were induced through a high-fat diet enriched with palmitic acid and/or endotoxin. The administration of amitriptyline protected the mice against body weight gain and ameliorated insulin signaling, as revealed by concomitant reductions in the circulating concentrations of insulin and decreased HOMA-IR (95). The administration of this drug also inhibited hepatic steatosis, hepatocellular ballooning, and hepatic inflammation. Noteworthy is that on isolated macrophages from these mice, amitriptyline successfully suppressed SP1 formation, reduced ceramide content, and reduced pro-inflammatory cytokines such as IL-6 (95). In an independent study, the use of amitriptyline in wild-type mice fed a high-fat diet successfully reduced hepatic levels of ceramides. This reduction protected the development of hepatic steatosis, fibrosis, and liver inflammation induced by a high-fat diet (96). The hepatic content in ceramides was not eventually determined; it could be postulated that ceramides would be decreased in the treated mice. In support of this hypothesis, the treatment of wildtype mice with imipramine, another tricyclic antidepressant, reduced hepatic ceramides, which was accompanied by decreased steatosis and improved blood glucose tolerance (92). Concurrently, the administration of this drug was also able to reduce the ethanol-derived activation of stress kinases. Further research is needed, but imipramine could be improving MASH also in non-alcoholic animal models.

Another molecule identified as aSMase’s functional direct and high-affinity inhibitor is 1-aminodecylidene bis-phosphonic acid (ARC39), a bisphosphonate compound. ARC39 acts on the lysosomal and secretory forms of the enzyme and leads to a marked decrease of cellular ceramide in different hepatocyte cell types, i.e., L929, HepG2, and B16F10 cells. However, when administered intraperitoneally *in vivo*, ARC39 showed renal and hepatic toxicity, whereas lower doses, not related to adverse toxic effects, did not influence ceramide content (94). Further research may still be needed to assess the efficacy of this drug *in vivo*.

#### 4.1.5 Specific inhibitors of dihydroceramide desaturase 1

Another possible therapeutic target is the enzyme dihydroceramide desaturase 1 (DES1), which is involved in ceramide production (116). The synthetic retinoid Fenretinide acts as a DES1 antagonist, inhibiting ceramide *de novo* synthesis. In RAW 264.7 macrophage cells, Fenretinide decreased ceramide level and inhibited the release of proinflammatory cytokines (99). In a mouse model of atherosclerosis and MASLD, Fenretinide prevented body weight gain, and improved the signs of MASLD, while reducing the gene expression of fibrosis markers. However, a worsening in aortic plaque formation was observed (100).

In lung epithelial cells, inhibition of DES1 by XM462 led to protection from the inflammatory effects of smoke, reducing several inflammatory cytokines (101). In an obese model of leptin-deficient mice, whole-body DES1 deletion resolved hepatic steatosis and normalized insulin resistance. Moreover, liver-specific deletion of DES1 also led to the same results and decreased several inflammatory cytokines (102).

#### 4.1.6 Intestinal farnesoid X receptor antagonists

Accumulating experimental evidence recently accounted for intestinal farnesoid X receptor (FXR) as another potential candidate target to protect against MASLD/MASH (117). For instance, abrogating the FXR signaling with specific antagonists ameliorates MASLD and decreases circulating ceramide levels in high-fat-fed mice. Improved MASLD was explained by a decreased *de novo* lipogenesis in treated mice (118). Glycine-β-muricholic acid (Gly-MCA) is an FXR agonist that suppresses ceramide synthesis-related genes, thus reducing ceramide levels in the livers of treated mice (97). A generally improved inflammatory response was seen in both MASLD and MASH mice models treated with this agonist, which showed fewer injury markers and lower levels of aspartate and alanine transaminases (97). The reduction of ceramide levels was concomitant with a lower liver ER stress and reduced proinflammatory cytokine production, which accounted for the much lower degree of inflammation in the livers of treated mice (97).

More recently, in patients with suspected fibrotic MASH, vonafexor, a second-generation, non-bile acid farnesoid X receptor agonist, markedly reduced liver steatosis and showed an improvement in imaging biomarkers of fibrotic steatohepatitis after only 12 weeks of treatment (98).

#### 4.1.7 Other approaches

Food supplements and dietary manipulations are another potential strategy to influence sphingolipid alterations and prevent MASH development. A high intake of vegetables and fruits directly affects circulating ceramide levels, as shown in two independent studies. The first was a sub-study of the PREDIMED trial in 980 subjects following a Mediterranean diet, which showed significantly lower circulating levels of ceramides than the control group (119). Similarly, circulating ceramides were also lower in another study, reducing general circulating ceramide content but C16:0 and lowering general inflammatory status (120). Another clinical trial observed that subjects eating a diet high in unsaturated fatty acids had reduced plasmatic fatty acids containing sphingolipids than subjects eating a diet high in saturated fatty acids (103). However, none of these human studies analyzed hepatic status.

Regarding dietary supplements, in a study performed on lean female rats, green coffee extract reduced hepatic triglyceride content and C20:0 ceramide (121). Another study using scoparone, a compound derived from the *Artemisia capillaris* plant used in traditional Chinese medicine as a lipid-lowering and anti-inflammatory (122), in a MASLD mice model fed a high-fat diet, the authors observed that the sphingolipid profile was favorably modified, decreasing the levels of several ceramides upon scoparone treatment (104).

### 4.2 Anti-diabetics

Among antidiabetic pharmacological agents currently used to treat T2D, only two drug groups, i.e., thiazolidinediones (pioglitazone) and GLP-1 receptor agonists, but not metformin, have been shown to alleviate MASH (123, 124). Apart from these, despite favorably influencing hepatic steatosis, no clinical evidence has been reported on MASH amelioration by other frequently used antidiabetic drugs, i.e., sodium–glucose cotransporter 2 (SGLT2) inhibitors and insulin.

As traditional antidiabetic interventions continue to evolve, a deeper understanding of their influence on MASLD/MASH, and more specifically, sphingolipid pathways, holds the potential to reveal novel insights into disease mechanisms and therapeutic strategies. Among the various antidiabetic medications, each demonstrates distinctive impacts on lipid profiles, explicitly targeting ceramides, phosphatidylcholines, SMs, and triglycerides, suggesting a broader association between antidiabetic therapies and lipid metabolism modulation. **Table 2** summarizes the evidence regarding the impact of the most studied antidiabetic drugs on sphingolipid metabolism, exploring their potential connection to MASLD.

**Table 2:** The most researched antidiabetic drugs on sphingolipid metabolism and their potential connection to MASLD*.

Drug		Therapeutic action	Sphingolipid metabolism		MASLD impact
			Experimental	Clinical	
Metformin		Suppressed liver gluconeogenesis, decreased intestinal absorption of glucose, and improved insulin sensitivity (125).	Promotes mitochondrial β-oxidation, decreasing ceramides and diacylglycerol levels (76, 126, 127).	Reduced levels of 3 ceramides and 4 sphingomyelins in subjects with PCOS. Orally, 4 week progression from 500 mg/day to 1500 mg/day, dose continued during 7 weeks (128). Decreased the levels of S1P after a single oral dose of 500 mg metformin (129).	Reduction of ceramides redirecting FA metabolism from energy storage to expenditure. Human hepatocytes were treated with 1 mM metformin (127). May reduce the incidence and death rate of NASH-related HCC (130).
Thiazolidinediones	Pioglitazone	Improved insulin sensitivity and binding to the peroxisome proliferator-activated receptor gamma (PPARγ) (125).	Reduction in certain ceramide species (C22:1, C23:0) and intrahepatic diacylglycerol. Orally through diet supplemented with pioglitazone (0.01%) (131). No changes in ceramides in subjects with T2D and obesity. Pioglitazone was administered orally for 2 or 7 weeks (2.5 mg/kg) (132, 133).	Reduced ceramide concentrations in individuals with metabolic syndrome. Pioglitazone orally administered (45mg/day) (134). No changes in ceramides in subjects with T2D and obesity but increased saturation of FA within cell membrane lipids. Pioglitazone was administered orally for 6 months (45mg/day) (132).	Improvement in individual histologic scores, adipose tissue, hepatic, and muscle insulin sensitivity (125).
	Rosiglitazone		No changes in ceramide levels. Mice were fed rosiglitazone mixed with chow diet (200 mg/kg chow) for 20 or 28 days, or (50 ppm) for 8 weeks, respectively (135, 136).	Limited evidence; further research needed.	Improvement of steatosis correlated with a reduction of transaminase level improvement in insulin sensitivity. No results on ballooning and fibrosis (125).
GLP-1 receptor agonists	Liraglutide	Enhanced glucose-dependent insulin secretion; inhibition of glucagon release from the pancreas (125).	Prevents accumulation of C16 and C24-ceramides. Liraglutide was infused via micro-osmotic pump (0.1 µl/h) (137).	Reductions in ceramides, hexocyl-ceramides, phosphatidylcholines, and triglycerides. Liraglutide was administered orally at a starting dose of 0.6 mg/day and up to 1.8 mg/day maintenance-dose during 26 weeks (138). Reduction in specific ceramides associated with CVD risk (C16 Cer and C24:1 Cer). Liraglutide was administered orally (1.8 mg/day) during 12 weeks (139).	Prevention of ceramide accumulation in cardiac progenitor cells, and displayed anti-steatotic effects. 31% reduction of hepatic steatosis (125). Significant reductions in ceramides (18:0, 18:1, 19:0, 24:1 and 26:1) and decreased liver fat content (p=0.0005). Subjects receives liraglutide (1.2 mg/day) for 6 months (140).
	Exenatide		Decrease in diacylglycerols and ceramides. Mice were daily injected with exenatide for 8 weeks (30 µg/kg of body weight) (141).		Reduced steatosis, significantly decreased NASH-related biomarkers (ALT, AST, GGT). Improved fibrosis score (NFS, APRI score but not FIB-4). Exenatide was given orally at a dose of 10 μg per day the first 4 weeks, and continued up to 6 months at 20 μg per day (142). Decrease in ceramides. Downregulation in hepatic lipogenic genes and genes involved in inflammation and fibrosis (141).
SGLT2 inhibitors	Empagliflozin	Increased glucagon levels, reduced renal reabsorption of glucose increasing its excretion (125).	Reduced SM, ceramide, S1P, and neutral CDase activity in diabetic rats. Empagliflozin was daily administered (30 mg/kg) during 4 weeks (143).	Change in sphingosine/ceramide metabolism via neutral CDase in individuals with T2D. Subjects received empagliflozin (25 mg/day) for 4 weeks (144).	Reduction in BMI, cholesterol, GGT, ballooning, and fibrosis. Improvement in liver steatosis and serum ALT level (125). Lowered hepatic lactosylceramides (LCER 16:0, 18:0). Mice received oral empagliflozin for 12 weeks 10 mg/kg/day) (145)
GIP and GLP-1 receptor agonist	Tirzepatide	Stimulated insulin release from the pancreas and increased adiponectin levels (146).	Limited evidence; further research needed.	Reduced levels of individual saturated ceramides and sphingomyelins; increased levels of individual unsaturated sphingomyelins and conjugated ceramides. Subjects received subcutaneously doses of tirzepatide (1, 5, 10, 15 mg) once a week for 26 weeks (147).	Significantly decreased NASH-related biomarkers (ALT, cytokeratin-18, procollagen III) and increased adiponectin in patients with T2D. Patients received subcutaneously doses of tirzepatide (1, 5, 10, 15 mg) once a week for 26 weeks (148).

MASLD, metabolic associated steatotic liver disease FA, fatty acids; NAFLD, non-alcoholic fatty liver disease; HCC, hepatocellular carcinoma; CVD, cardiovascular disease; T2D, type 2 diabetes; BMI, body mass index; GGT, gamma-glutamyl transferase; ALT, alanine aminotransferase; GIP, gastric inhibitory polypeptide.

*When indicated, the current terms MASH/MASLD that appear in this table were used instead of the traditional terms NASH/NAFLD.

#### 4.2.1 Metformin

Metformin demonstrated efficacy in blocking the increase of fatty acid transport protein CD36, along with aberrant ceramide and diacylglycerol content in the skeletal muscle of diabetic rats (76). Further experimental evidence also suggests that metformin’s insulin-sensitizing effect in the liver promotes mitochondrial β-oxidation, protecting against ceramides and diacylglycerol accumulation and preserving insulin sensitivity under a high-fat diet consumption (126).

Clinical studies support this evidence. For instance, the lipidomic profile of 27 individuals with polycystic ovary syndrome (PCOS) revealed lower levels of three ceramides and four SM after treatment with metformin. This suggests that this antidiabetic drug may influence insulin resistance in women with PCOS by interacting with ceramides, critical players in the insulin signaling pathway (149). In healthy subjects, metformin significantly decreased the levels of S1P, a bioactive lipid generated by converting ceramide to sphingosine. S1P is associated with inflammation, immunity, and insulin resistance and has also been associated with the development of obesity and T2D (129). The changes in lipid species indicated essential lipid signaling pathways that might be related to the varied effects of metformin.

An experimental study with human hepatocytes aimed to elucidate the pathogenesis of MASLD. It found significant changes in genes related to fatty acid metabolism using metformin as a steatosis inhibitor. These changes include a reduction of ceramides, higher mitochondrial activity, and enabled β-oxidation, redirecting fatty acid metabolism from energy storage to expenditure (127).

#### 4.2.2 Thiazolidinediones

Pioglitazone, a thiazolidinedione primarily targeting the PPARγ receptor, has demonstrated efficacy in improving the liver histology of non-diabetic non-alcoholic steatohepatitis subjects in clinical trials (150). Experimental models, particularly a mouse model simulating non-alcoholic steatohepatitis, linked pioglitazone to enhanced hepatic mitochondrial function, coupled with a significant decrease in intrahepatic diacylglycerol classes and specific ceramide species (C22:1, C23:0) (131).

In individuals with metabolic syndrome, a study indicated that pioglitazone significantly reduced multiple plasma ceramide concentrations, aligning with improvements in insulin resistance and adiponectin levels (134). However, a clinical study including patients with diabetes and obesity following six months of pioglitazone treatment (45 mg/day) did not show significant changes in the ceramide or diacylglyceride content in adipose tissue. Instead, pioglitazone induced a selective remodeling of the glycerophospholipid pool, resulting in increased overall saturation and shortened chain length of fatty acyl groups within cell membrane lipids (132). Other studies align with these results (133), underscoring the need for further research to elucidate lipid metabolism and pioglitazone treatment.

Experimental studies have shown no changes in ceramide levels relating to rosiglitazone treatment (135, 136).

#### 4.2.3 Glucagon-like peptide-1 receptor agonists

Liraglutide, a glucagon-like peptide-1 receptor agonist (GLP-1 RA), has significantly impacted lipid metabolism. In the LiraFlame trial, involving 102 participants with T2D, liraglutide treatment for 26 weeks led to substantial reductions in ceramides, hexocyl-ceramides, phosphatidylcholines, phosphatidylethanolamines, and triglycerides (138). Furthermore, a postHOC of two randomized control trials (RCTs) found that in the LiraFlame26 trial, liraglutide significantly reduced specific ceramides associated with cardiovascular risk, namely C16 and C24:1, compared to placebo. Conversely, in the LirAlbu12 trial, a 12-week liraglutide treatment did not result in significant ceramide changes. Weight loss did not affect the observed results (139).

Regarding insulin resistance and liver steatosis, in patients with T2D, six months of treatment with liraglutide (1.2 mg/day) led to a significant decrease in total dihydroceramide by 15.1%, affecting 16:0, 18:0, 18:1, 20:0, 23:0 and 24:1 species. Total plasma ceramides did not significantly change after treatment, but species 18:0, 18:1, 19:0, 24:1 and 26:1 decreased significantly. The reduction in dihydroceramide after liraglutide was independently associated with reduced liver fat content (p=0.0005) and improved insulin resistance measured by the TyG index (*p*-value=0.05) (140). Other studies exploring liraglutide’s impact in MASLD demonstrated the potential to down-regulate genes related to glycoceramide metabolism, prevent ceramide accumulation in cardiac progenitor cells, and offer anti-steatotic effects (151). Overall, the evidence supports the role of liraglutide in lipid metabolism modulation, providing potential therapeutic benefits in conditions like T2D, MASLD, and cardiovascular disease.

On the other hand, exenatide, another GLP-1 RA, has shown significant improvements in lipid metabolism, as demonstrated in a diet-induced mouse model of MASH. The study revealed that exenatide significantly decreased intrahepatic triglyceride content, reduced 23% hepatic glucose production, reduced insulin resistance, and alleviated hepatocyte lipotoxicity. The lipidomic profile showed decreased diacylglycerols and ceramides, downregulated hepatic lipogenic genes, and genes involved in inflammation and fibrosis (141).

With regard to other GLP-1 RA, in a randomized controlled trial (RCT) comparing dulaglutide with another drug and placebo, a lipidomic analysis found no changes in total ceramides and SM (147).

#### 4.2.4 Sodium-glucose cotransporter 2 inhibitors

Research on empagliflozin suggests that SGLT2 inhibitor modulates sphingolipid metabolism, influencing *de novo* and catabolic pathways in diabetes and specifically downregulating the catabolic pathway in hypertension (143). In diabetic rats, empagliflozin treatment reduced SM, ceramide, S1P, and neutral CDase activity. In hypertensive rats, it decreased SM, S1P, aSMase, and neutral CDase activity (143). Moreover, in a study with diet-induced obese-MASH mice, empagliflozin treatment lowered hepatic lactosylceramides (LCER), including LCER (16:0) and LCER (18:0). Increased concentrations of unsaturated triglyceride species were observed, potentially contributing to improved hepatic inflammation through enhanced autophagy (145). Additionally, proteomic analysis of individuals with diabetes receiving a 4-week empagliflozin treatment (25 mg/day) led to significant alterations in 43 proteins, including neutral CDase. This change is related to the sphingosine/ceramide metabolism, a known pathway of cardiovascular disease (144).

The role of SGLT-2 inhibitors, including empagliflozin, dapagliflozin, and ipragliflozin, in protecting against MASLD progression involves decreasing *de novo* lipogenesis, fatty acid uptake, and hepatic triglyceride secretion while promoting vital regulatory genes of fatty acid β-oxidation (152).

#### 4.2.5 New antidiabetic drugs

Experimental studies revealed that treating prediabetic rats with pioglitazone and alogliptin prevents diabetes onset and lowers islet lipids. However, they did not fully restore islet function or lower ceramide levels (133). Moreover, even though its usage in diabetes is in decay, acarbose significantly influenced fatty acids and sphingolipids in the liver, leading to notable changes in more than ten species (some increased while others decreased) in experimental studies (153).

In the clinical setting, a study comparing lipid profiles of patients treated with glipizide or metformin found significant increases in both groups in phosphatidylcholine (PC) lipids and decreases in SM species. Of these, PC (O-34:1), SM (d18:0–24:1), and SM (d18:1–20:1) were associated with long-term composite cardiovascular events. Lipidomic data revealed alterations in 7 lipid classes, including SM, known for their roles in diabetes and atherosclerosis (154).

Similarly, in a study of the metabolomic profile of subjects treated with liraglutide or glimepiride, significant effects on various lipid classes were observed, including free fatty acids, amino acids, bile acids, triglycerides, phosphatidylethanolamines, and SM. Particularly, substantial declines in SM (d33:1), SM (40:2), and SM (37:1) were noted following the administration of glimepiride (155). More recently, in a lipidomic analysis of an RCT, tirzepatide, a dual glucose-dependent insulinotropic polypeptide (GIP) and GLP-1 RA, lowered levels of individual saturated ceramides and SM and increased levels of individual unsaturated SM and conjugated ceramides. No changes in total ceramides and SM were observed (147).

Future studies may further elucidate the mechanisms through which new antidiabetics benefit patients with metabolic disorders. Reducing lipotoxic lipids with antidiabetic drug therapy could pave the way toward precision medicine.

## 5 Conclusions and perspectives

There are no specific non-invasive biomarkers for MASH diagnosis/prognosis. Though liver biopsy is considered the gold standard for diagnosis of MASLD in subjects at very high risk, the dramatic rise in the prevalence of this condition and that of its advanced form, i.e., MASH, means that such a histological approach has become limited in clinical practice. In this context, clinicians have limited the assessment of fibrosis using imaging techniques, such as transient elastography, in combination with serum surrogate biomarkers of inflammation and fibrosis to identify subjects at high risk for MASH. In the era of “omics”, the use of novel, innovative approaches may not only lead to uncover hidden biomarkers but also allow better understanding of the pathophysiology of this condition. Notably, lipid metabolism, including that of sphingolipids, is profoundly altered in MASLD/MASH. Experimental and recent clinical studies suggest that some sphingolipid species, especially ceramides and dihydroceramides, are differentially expressed in subjects with MASH compared with MASLD and non-steatotic livers. A similar differential sphingolipid pattern has also been identified in paired serum samples. As the liver is considered the main contributor to circulating ceramides and, conceivably, dihydroceramides, their direct determination in serum samples may help open novel avenues in MASH diagnosis.

The assessment of novel experimental drugs targeting key enzymes involved in ceramide production are under intense investigation for combating MASH. Noteworthy, recent research has also unveiled the repurposing potential of “old” drugs with a “new dress”. For instance, fingolimod, an approved treatment for multiple sclerosis, and some frequently used antidiabetic drugs, such as pioglitazone and GLP-1 RA, have demonstrated efficacy in protecting against MASH development through favorably influencing sphingolipid metabolism in experimental animal models. Overall, pharmacological targeting of sphingolipid metabolism could open a promising new therapeutic strategy for patients with this chronic liver condition.

## Author contributions

BR: Conceptualization, Supervision, Writing – original draft, Writing – review & editing. JR: Conceptualization, Supervision, Writing – original draft, Writing – review & editing. AP: Conceptualization, Supervision, Writing – original draft, Writing – review & editing. EP: Writing – original draft, Writing – review & editing. IG: Writing – original draft, Writing – review & editing. MR: Writing – review & editing. MJ: Writing – review & editing. NA: Funding acquisition, Writing – review & editing. JJ: Conceptualization, Funding acquisition, Supervision, Writing – original draft, Writing – review & editing. DM: Conceptualization, Supervision, Writing – original draft, Writing – review & editing.
